# Prehabilitation in Frail Octogenarian and Nonagenarian Patients in Colorectal Cancer Surgery: Short- and Medium-Term Outcomes

**DOI:** 10.3390/jcm13206114

**Published:** 2024-10-14

**Authors:** Raquel Ramírez-Martín, Coro Mauleón Ladrero, Jose Antonio Gazo Martínez, Victoria Déniz-González, Isabel Martín Maestre, Lucía Corral-Sastre, María Villajos-Guijarro, Rocío Menéndez-Colino, Isabel Pascual Miguelañez, Juan Ignacio González-Montalvo

**Affiliations:** 1 Department of Geriatric, Hospital Universitario La Paz, 28046 Madrid, Spain; mariadelcoro.mauleon@salud.madrid.org (C.M.L.); victoriamaria.deniz@salud.madrid.org (V.D.-G.); marmaeis@gmail.com (I.M.M.); lucia771@hotmail.com (L.C.-S.); mariavillajos@gmail.com (M.V.-G.);; 2 Hospital La Paz Institute for Health Research—IdiPAZ, Hospital Universitario La Paz—Universidad Autónoma de Madrid, 28046 Madrid, Spain; 3 Department of General Surgery, Hospital Universitario La Paz, 28046 Madrid, Spain; 4 Medicine Department, School of Medicine, Universidad Autónoma de Madrid, 28049 Madrid, Spain

**Keywords:** prehabilitation, frailty, nonagenarian, octogenarian

## Abstract

**Background:** There is still limited evidence on the results of prehabilitation in very old frail patients. The aim of this study is to analyze the outcomes and course of octogenarian and nonagenarian patients undergoing prehabilitation before surgery for colorectal cancer (CRC). **Methods**: a prospective study was conducted in a tertiary hospital from 2018 to 2022. All patients diagnosed with CRC over 80 years old and proposed for surgery were included. A comprehensive geriatric assessment (CGA) for frailty detection was performed, and the therapeutic decision was taken by the multidisciplinary tumor committee. Prehabilitation led by the geriatric team was performed. The rate of medical and surgical complications, hospital stay, in-hospital mortality, and first-year mortality were recorded. **Results**: CRC surgery was proposed in 184 patients >80 years. After a multidisciplinary decision, surgery was performed on 126 (68.5%) patients, of whom 12 (0.5%) were nonagenarians. Fifty percent of octogenarians and 86% of nonagenarians were frail. Prehabilitation consisted of the following: adapted physical exercise (100%); oral nutritional supplementation (73.8%); anemia treatment (59.5%); delirium prevention (5.6%); antidepressant treatment (15.9%); vitamin D supplementation (21.4%); and pharmacological deprescription (38.1%). The post-surgical complication rate was low (4.3% surgical and 29.4% medical complications), and in-hospital mortality was very low (3.2%). Nonagenarian patients had a higher rate of complications compared to octogenarians (OR 4.0 (95% CI 1.13–14.12))—mainly heart failure (OR 4.68 (95% CI 1.21–18.09))—but there were no differences in hospital stay or first-year mortality. **Conclusions**: prehabilitation in very old patients with CRC surgery is possible and provides good results.

## 1. Introduction

Colorectal cancer (CRC) is one of the most frequent tumors, being the second most common in terms of incidence (10.2%) and the third most common in terms of mortality (9.2%) [1]. Globally, a 70% increase in CRC diagnoses is expected by 2045, and the population group in which the incidence of CRC will increase the most is the over-75s, with an increase of 164% [2]. Specifically, Spain has the third highest incidence of CRC in the older population worldwide [3]. Despite the high incidence of CRC in older adults, they are often under-represented in clinical trials [4] and the probability of receiving any specific cancer treatment for CRC decreases significantly [5]. Twenty-one per cent of patients over 85 years of age diagnosed with CRC are not operated on, while rates of non-surgical intervention in younger patients are lower [6]. This undertreatment of CRC impacts on mortality: the 3-year survival rate in >70 year old with untreated colon cancer is 9% compared to 91% in those who undergo surgery [7]. Studies show that the long-term prognosis for older CRC patients who survive the first year of surgery is similar to that of younger adults [8].

The Healthcare Cost and Utilization Project Nationwide Inpatient Sample, which analyzed more than one million patients who had undergone bowel resection for CRC, found that most of them (64%) were over 65 years old, and 23% were octogenarians and nonagenarians [9]. This increase in the number of older patients undergoing complex surgery is a challenge because these patients have a higher rate of post-surgical complications [10] and a higher postoperative mortality (6.2 times higher 30-day mortality in patients over 85 years of age compared to younger patients) [6].

Because of the increase in the number of older patients undergoing surgery for CRC, several guidelines published in recent years recommend that chronological age should not be the sole factor in decision-making; frailty screening should be performed. Additionally, frail patients benefit from a multidisciplinary approach to perioperative care, with a team including a healthcare professional with geriatric expertise [11,12]. This makes the comprehensive geriatric assessment (CGA) a cornerstone in the pre-surgical care of these patients, in which the geriatric team has two main functions: to assess the patient’s physiological reserve in helping decide on the best treatment as part of a multidisciplinary tumor committee and to collaborate in the prehabilitation of these patients. Optimization of the patient during the preoperative period with the aim of improving the postoperative outcome has been included in the concept of prehabilitation. In prehabilitation programs, actions such as improving nutritional status or physical performance can improve patients’ prognosis [13].

In recent years, numerous studies have analyzed the role of prehabilitation in older patients undergoing surgery for CRC [14,15,16,17,18,19,20,21,22,23,24]. However, some of these studies mix results of prehabilitation in CRC with other pathologies [15,16,22,25,26] or include patients undergoing emergency surgery [18,24]. In most studies, prehabilitation is exclusively physical (unimodal) [16,22] or trimodal (nutrition, exercise, and psychological intervention) [14,15,17,19,20,24], and in only four studies is prehabilitation multimodal and comprehensive, led by teams of geriatricians [25,26,27,28]. Moreover, although there are studies focusing on older people, only two include very old patients, with a mean age > 80 years [18,23]. There are studies in the literature that analyze the clinical course of nonagenarian patients undergoing surgery for CRC [29,30] but none analyze prehabilitation in nonagenarian patients.

Therefore, although there is already evidence that prehabilitation is effective in older patients with CRC, there are no studies that have examined prehabilitation in nonagenarian patients. The aim of this study is to analyze the effect of CGA and prehabilitation in nonagenarian patients undergoing surgery for CRC and to compare their course with octogenarian patients undergoing surgery for the same pathology.

## 2. Materials and Methods

The study included all patients aged 80 years or older with a new diagnosis of colon cancer or rectal cancer proposed for surgery at the Hospital Universitario La Paz (a 1000-bed teaching hospital). The patient inclusion period was from 1 October 2018 to 1 November 2022. The variables included in the study and the program carried out in these patients are already described in detail in a previous publication of this working group [31].

### 2.1. Preoperative Phase

#### 2.1.1. Evaluation at the Cross-Specialty Geriatrics Consultation

After being considered for surgery by the General Surgery Department, patients were evaluated in a geriatric consultation by a geriatrician and a geriatric nurse who performed the CGA. The data recorded were age, sex, comorbidity (Cumulative Illness Rating Scale-Geriatric (CIRS-G)) [32], usual pharmacological treatment, anesthetic risk (American Society of Anesthesiologists (ASA) scale) [33], functional status (Functional Ambulation Categories (FAC) [34], the Barthel Index [35] and the Lawton and Brody Scale) [36], cognitive status (Reisberg Global Deterioration Scale [37], and the Pfeiffer Short Portable Mental Status Questionnaire) [38]. Regarding nutritional assessment, body mass index (BMI), the Mini Nutritional Assessment Short Form (MNA-SF) [39], and serum proteins and serum albumin were recorded. Frailty status was assessed with gait speed (meters/s), manual handgrip strength (using the Jamar© hydraulic dynamometer), the Short Physical Performance Battery (SPPB) [40], the Clinical Frailty Scale [41,42], the Frail Scale [43], and the Frail-VIG Index [44]. In addition, hemoglobin, iron profile, and vitamin D were collected.

Comorbidity was considered severe in patients who had three or more points in two or more systems on the CIRS-G scale. High anesthetic risk was considered to be ASA ≥ III. The cut-off points for frailty detection following the various scales were as follows: gait speed ≤ 0.8 m/s, handgrip strength ≤ 12 kg in women and ≤22 kg in men [45], SPPB ≤ 9, Clinical Frailty Scale ≥ 5, Frail Scale ≥ 3, and Frail-VIG Index ≥ 0.2.

#### 2.1.2. Prehabilitation and Preoperative Geriatric Intervention

All patients in the study received an individualized geriatric intervention based on the deficits detected in the comprehensive assessment. After a pharmacological review, potentially inappropriate drugs were deprescribed. Oral protein supplementation was prescribed to patients with evidence of malnutrition (BMI < 22 kg/m^2^, weight loss >5% in the last month, MNA-SF ≤ 11 points, hypoproteinaemia (protein ≤ 6 g/dL) or hypoalbuminemia (serum albumin ≤ 3 g/dL)). Multicomponent physical exercise adapted to their situation was prescribed on an individual basis, using the ViviFrail program [46]. Patients with iron deficiency (ferritin < 100 ng/mL or transferrin saturation index <20%) were treated with intravenous iron. In case of deficiency (<30 ng/mL), vitamin D supplementation was prescribed. In case of social frailty, an assessment by the social work team was requested.

#### 2.1.3. Multidisciplinary Clinical Decision

The cancer treatment decision was decided in a multidisciplinary manner in the colorectal Tumor Committee after evaluation of the patient in the Geriatrics Department and also taking into account the patient’s wishes and preferences. This multidisciplinary committee was made up of general surgeons, radiation and medical oncologists, pathologists, radiologists, gastroenterology, nuclear physicians, and geriatricians.

### 2.2. In-Hospital Phase: Follow-Up during Admission

Cases of emergency surgery were excluded. Patients who underwent scheduled CRC surgery received daily clinical follow-up by the geriatrician in addition to the usual care provided by the General Surgery Service. During the days of admission, the presence of medical or surgical complications (such as pneumonia, urinary tract infection, atrial fibrillation, acute heart failure, pulmonary thromboembolism, bacteraemia, delirium, surgical wound dehiscence or infection, surgical reintervention, readmission to the anesthesia resuscitation unit, and stay in the anesthesia resuscitation unit > 48 h) were recorded. In addition, degree of severity of complications (Clavien-Dindo [47] ≥ Grade III), in-hospital stay, and mortality were recorded. The diagnosis of delirium was made according to the criteria defined by the Diagnostic and Statistical Manual of Mental Disorders, fifth edition.

### 2.3. Post-Discharge Follow-Up Phase

Follow-up was carried out for 12 months after hospital discharge, recording mortality at 30 days, 6 months, and 12 months and at readmissions or visits to the emergency department in the first month.

The statistical analysis was performed using the SPSS Version 17 statistical software package. A descriptive analysis of the variables was carried out (mean and standard deviation for quantitative data and absolute and relative frequencies for qualitative data). The percentages for each variable out of the total valid data for each variable are presented. Statistical comparisons of the parameters evaluated were performed using parametric tests (Student’s *t*-test and analysis of variance), except when selection or stratification required nonparametric tests (Kruskal–Wallis and Mann–Whitney U test). The Kaplan–Meier method was used to estimate survival. It was considered statistically significant if *p* < 0.05.

This study was approved by the Clinical Research Ethics Committee of the Hospital Universitario La Paz (PI-3729), and all participants gave informed consent.

## 3. Results

During the 49 months of the study, 184 patients with a diagnosis of CRC who were candidates for surgery were included. Of these, 160 (87%) were octogenarians and 24 (13%) were nonagenarians. The clinical characteristics of the patients and the differences by age (octogenarians or nonagenarians) are shown in Table 1. Nonagenarian patients were more often female, had higher rates of polypharmacy, anemia, and frailty, as measured by SPPB, gait speed, and the Clinical Frailty Scale.

Geriatric intervention was performed on all the patients. A multimodal prehabilitation led by the geriatrics team was carried out, in which the most frequent interventions were as follows: adapted physical exercise (100%); oral nutritional supplementation (73.8%); anemia treatment (59.5%); delirium prevention (5.6%); antidepressant treatment (15.9%); vitamin D supplementation (21.4%); and pharmacological deprescription (38.1%). There were no differences in the prehabilitation interventions between the two groups of patients (*p* > 0.05)

**Figure 1:**
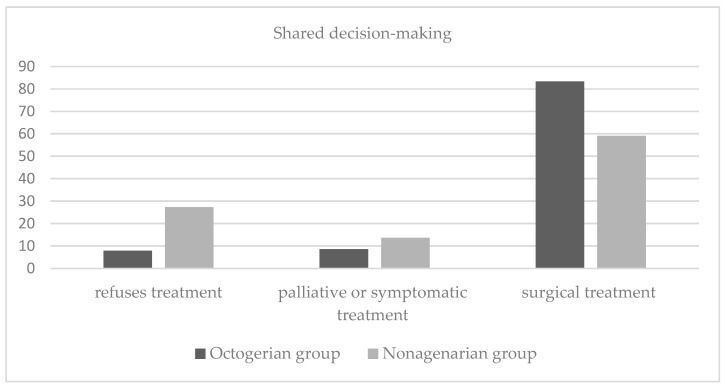
Therapeutic decision-making in colorectal cancer as a function of age for octogenarians and nonagenarians.

Decision-making was shared in the multidisciplinary committee for colorectal tumors, taking into account the patient’s values and preferences. Compared to younger patients, nonagenarian patients had a lower rate of surgical intervention (83.4% vs. 59.1%) and a higher rate of refusal of treatment (7.9% vs. 27.3%) *p* = 0.03 (Figure 1).

Of the 134 patients scheduled for surgery, eight patients (seven octogenarians and one nonagenarian) required urgent surgery during the surgical waiting period, resulting in 126 patients (114 octogenarians and 12 nonagenarians) undergoing scheduled surgery.

**Figure 2:**
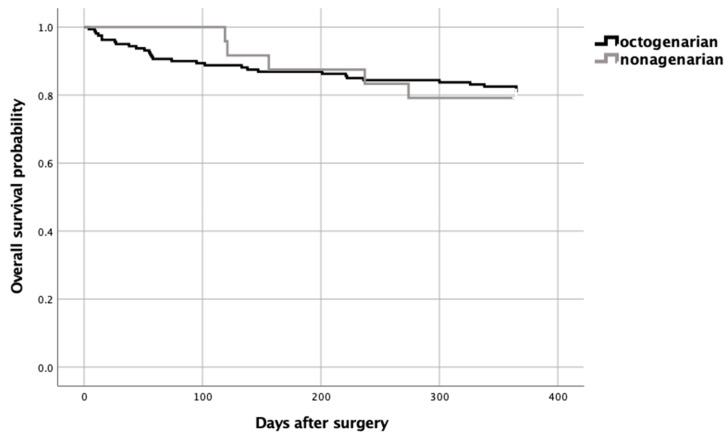
Kaplan–Meier-estimated overall survival curves for octogenarian and nonagenarian patients who underwent surgery for colorectal cancer and had previously undergone prehabilitation.

Nonagenarian patients who underwent surgery had a higher frequency of complications (OR 4.0 (95% CI 1.13–14.12))—mainly heart failure (OR 4.68 (95% CI 1.21–18.09))—compared to octogenarian patients. But there were no differences in hospital stay, surgical complications, or in-hospital mortality. There was also no difference in 30-day hospital readmissions or mortality during the first year compared to octogenarian patients (Table 2 and Figure 2).

**Figure 3:**
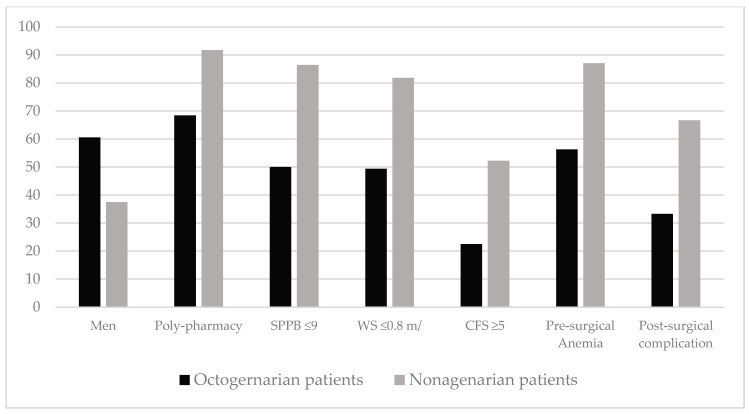
Statistically significant (*p* < 0.05) pre- and post-surgical differences between octogenarian and nonagenarian patients in prehabilitated patients undergoing colorectal cancer surgery. SPPB: Short Physical Performance Battery, WS: Walking Speed, CFS: Clinical Frailty Scale.

The statistically significant pre- and post-surgical differences between octogenarian and nonagenarian patients are shown in Figure 3.

## 4. Discussion

This study shows that nonagenarian patients selected through a comprehensive geriatric assessment and subjected to a prehabilitation program prior to scheduled surgery for CRC have a similar outcome and mortality to octogenarian patients undergoing surgery for the same pathology.

Nonagenarian patients with a diagnosis of CRC, despite presenting good functional and cognitive status, had a higher rate of polypharmacy, anemia, and frailty compared to octogenarian patients. This is why this subgroup of patients benefits even more from the care of the geriatric team, as greater complexity is involved in clinical decision-making and there are more areas that can be optimized with prehabilitation.

In addition, it is important to note that the patients were actively involved in the therapeutic decision. After being informed by the surgeon of the diagnosis, prognosis of their tumor, and therapeutic alternatives, and by the geriatrician of their fragility and prognosis for survival, they decided whether they wanted to undergo surgery. After receiving this information, 27.3% of nonagenarian patients refused treatment, while only 7.9% of octogenarian patients refused treatment. There are few studies analyzing the rate of treatment refusal after CRC diagnosis, although this decision is more frequent in older patients than younger ones and, according to a study published by Kaltenmeier et al., patients over 80 years of age account for more than 90% of treatment refusals, with an average treatment refusal rate of 2.5% [48]. In a recently published systematic review, the rates of refusal of surgery were similar, between 0.25 and 3.26% [49]. These figures contrast with those of our study, where the refusal rate was higher than that reported in the literature. This high refusal rate can be explained by the fact that the patients played an active part in the decision-making process, with the team avoiding paternalistic attitudes and discussing the individual situation in detail with them, taking into account the results of the comprehensive geriatric assessment, analyzing their physiological reserve and assessing the possible risks of surgery.

Although studies have analyzed the clinical course of nonagenarian patients undergoing surgery for CRC [29,30], there are no studies focusing on prehabilitation in these patients. Prehabilitation has been shown to lower the rate of post-surgical complications and the average hospital stay [50]. According to a recent meta-analysis, prehabilitation in frail patients undergoing surgery for CRC significantly reduces the incidence of post-surgical complications (OR 0.51, 95% CI 0.34–0.78) and mean hospital stay (OR −0.34, 95% CI −0.46–0.26) compared to frail patients managed without prehabilitation [51]. Although prehabilitation has been shown to be safe and efficient in older patients [52], there are still no studies that analyze its impact on the outcome of nonagenarian patients undergoing surgery for CRC.

In such patients, post-surgical complications are reported in 70% of cases, with an in-hospital mortality rate of 4.5–7.6% [29,30]. In our study, 66.7% of the nonagenarian patients had postoperative complications, with a risk of complications twice as high as in octogenarian patients, but only 10% had severe complications. Despite this, they did not have longer hospital stays, the mean stay being 9.2 (SD 4.6) days, similar to that of younger patients. Furthermore, in our study, in-hospital mortality was lower than in previous studies (4.5–7.6%) [29,30] and there were no in-hospital deaths among the nonagenarian patients who underwent surgery. Mortality during the first year after CRC surgery was also low, with a one-year survival rate of 83.3% and a survival curve overlapping that of younger patients.

All these data suggest that selected nonagenarian patients undergoing prehabilitation have a similar short- and medium-term clinical course to younger patients.

This study has several limitations that should be mentioned. Firstly, the sample size was relatively small and there were few fatal events, which may result in a lack of statistical confidence. Secondly, the rate of adherence to prescribed prehabilitation was not recorded. Thirdly, it should be noted that the care of these older patients was carried out in a tertiary hospital with considerable experience in the care of highly complex surgical patients and by a multidisciplinary team, so the results may not be generalizable to other hospitals. Fourthly, there is a limitation due to the design of the study as there is no control group.

Worth highlighting among its strengths are its representative population, including all patients over 80 years of age presented to the colorectal tumor committee during the study period in the reference hospital covering a health area of 520,000 inhabitants, and the low number of losses, thereby contributing to its population and epidemiological representativeness. Another strength is that a comprehensive geriatric assessment was performed with different frailty scales and geriatric-surgical co-management was performed during hospital admission.

Although clinical guidelines recommend that age alone should not be taken into account when making decisions, therapeutic abstention is still common at extreme ages of life, such as in nonagenarian patients. This study may provide an opportunity for future research by including nonagenarian patients in prehabilitation programs and involving geriatricians in multidisciplinary tumor committees.

## 5. Conclusions

In conclusion, elective colorectal cancer surgery in previously selected and prehabilitated nonagenarian patients was performed safely and with good short- and long-term results, with outcomes similar to octogenarian patients.

It is expected that in the coming years, due to an ageing population, the number of nonagenarian patients undergoing surgery for CRC will increase. This means that more scientific evidence in this subgroup of patients is required.

## Figures and Tables

**Table 1:** Baseline characteristics of patients with colorectal cancer in octogenarian and nonagenarian groups. Data are presented in *n* (%), except Functional Ambulation Categories and Barthel Index, which are expressed as the mean (SD).

Clinical Characteristics	Octogenarian Group N = 160	Nonagenarian Group N = 24	*p*
Men, *n* (%)	97 (60.6)	9 (37.5)	0.033
Severe comorbidity (CIRS-G ≥ 3 in ≥2 systems), *n* (%)	39 (24.4)	7 (29.2)	0.613
High anesthetic risk (ASA ≥3), *n* (%)	110 (82.7)	13 (92.9)	0.328
Polypharmacy (≥5 drugs), *n* (%)	108 (68.4)	22 (91.7)	0.018
Functional Ambulation Categories, mean (SD)	4.6 (0.9)	4.6 (0.6)	0.906
Barthel Index, mean (SD)	90.7 (17.5)	90.4 (8.3)	0.934
Global Deterioration Scale ≥ 3, *n* (%)	29 (18.2)	4 (16.7)	0.852
Nutritional status Body mass index ≤ 22 kg/m^2^, *n* (%) Mini Nutritional Assessment Short Form ≤ 11, *n* (%)	26 (16.6) 116 (72.5)	3 (12.5) 18 (75)	0.614 0.797
Frailty status Short Physical Performance Battery ≤ 9, *n* (%) Walking speed ≤ 0.8 m/s, *n* (%) Low handgrip strength, *n* (%) Clinical Frailty Scale ≥ 5, *n* (%) Frail Scale ≥ 3, *n* (%) Frail-VIG index ≥ 0.2, *n* (%)	80 (50) 79 (49.4) 61 (38.1) 36 (22.5) 29 (18.2) 64 (40.5)	19 (86.4) 18 (81.8) 13 (56.5) 12 (52.2) 6 (22.1) 10 (43.4)	0.001 0.004 0.093 0.002 0.371 0.786
Analytical parameters Anemia, *n* (%) Ferropenia, *n* (%) Vitamin D deficiency, *n* (%)	89 (56.3) 107 (89.2) 49 (86)	20 (87.0) 17 (81) 8 (72.7)	0.019 0.286 0.275

**Table 2:** Clinical outcomes of patients undergoing planned surgery for colorectal cancer: octogenarians and nonagenarians.

Clinical Outcomes	Octogenarian Group N = 114	Nonagenarian Group N = 12	*p*
Length of stay in days, mean (SD)	9.5 (6.9)	9.2 (4.6)	0.896
Surgical complications, *n* (%) Wound dehiscence Wound infection Surgical re-intervention ICU readmission ICU stay > 48 h	15 (13.2) 4 (3.5) 3 (2.6) 6 (5.3) 6 (5.3) 7 (6.1)	3 (25) 1 (8.3) 0 (0) 1 (8.3) 0 (0) 1 (8.3)	0.265 0.415 0.570 0.659 0.413 0.767
Medical complications, *n* (%) Heart failure Atrial fibrillation Respiratory infection Urinary tract infection Delirium Pulmonary thromboembolism Bacteremia	31 (27.2) 11 (9.6) 4 (3.5) 7 (6.1) 6 (5.3) 17 (14.9) 0 (0) 3 (2.6)	6 (50.0) 4 (33.3) 0 (0) 0 (0) 0 (0) 4 (33.4) 0 (0) 0 (0)	0.099 0.016 0.510 0.377 0.415 0.103 - 0.570
Any medical or surgical complication, *n* (%)	38 (33.3)	8 (66.7)	0.023
Clavien-Dindo ≥ 3, *n* (%)	4 (6.2)	4 (10)	0.713
In-hospital mortality, *n* (%)	4 (3.5)	0 (0)	0.510
30-day hospital readmissions, *n* (%)	9 (7.5)	0 (0)	0.557
6 month mortality, *n* (%)	12 (10.5)	1 (8.3)	0.812
12 month mortality, *n* (%)	15 (13.2)	2 (16.7)	0.735

## Data Availability

The original contributions presented in the study are included in the article, further inquiries can be directed to the corresponding author.
